# A Multiplex Label-Free Approach to Avian Influenza Surveillance and Serology

**DOI:** 10.1371/journal.pone.0134484

**Published:** 2015-08-04

**Authors:** Joseph Bucukovski, Neus Latorre-Margalef, David E. Stallknecht, Benjamin L. Miller

**Affiliations:** 1 Department of Biomedical Engineering, University of Rochester, Rochester, New York, United States of America; 2 Department of Dermatology, University of Rochester, Rochester, New York, United States of America; 3 Department of Population Health, College of Veterinary Medicine, University of Georgia, Athens, Georgia, United States of America; Icahn School of Medicine at Mount Sinai, UNITED STATES

## Abstract

Influenza serology has traditionally relied on techniques such as hemagglutination inhibition, microneutralization, and ELISA. These assays are complex, challenging to implement in a format allowing detection of several types of antibody-analyte interactions at once (multiplex), and troublesome to implement in the field. As an alternative, we have developed a hemagglutinin microarray on the Arrayed Imaging Reflectometry (AIR) platform. AIR provides sensitive, rapid, and label-free multiplex detection of targets in complex analyte samples such as serum. In preliminary work, we demonstrated the application of this array to the testing of human samples from a vaccine trial. Here, we report the application of an expanded label-free hemagglutinin microarray to the analysis of avian serum samples. Samples from influenza virus challenge experiments in mallards yielded strong, selective detection of antibodies to the challenge antigen in most cases. Samples acquired in the field from mallards were also analyzed, and compared with viral hemagglutinin inhibition and microneutralization assays. We find that the AIR hemagglutinin microarray can provide a simple and robust alternative to standard methods, offering substantially greater information density from a simple workflow.

## Introduction

Current methods of influenza serology including hemagglutination inhibition (HI), microneutralization (MN), and enzyme-linked immunosorbant assays (ELISA) have proven to be broadly useful in the clinical laboratory [1]. In the context of surveillance and evaluation of vaccine efficacy applications, however, the limitations of these assays including their complexity and ability to only provide information about a single antibody–antigen response at a time have proven problematic. Because of this, it is widely recognized that there is a need for new methods for detecting influenza antibodies [2]. Ideally, such technologies should be able to provide quantitative information about several antibody responses to different antigens simultaneously (i.e. a “multiplex” test) while doing so in a fast, reagentless, sample-conserving way (as, particularly for avian surveillance, limited volumes of sample are available), and independently of the host species tested. As alternatives to the traditional serologic assays, these new methods could dramatically simplify the process of analyzing samples acquired in the field.

We have recently developed a technology that should prove useful in addressing this goal. Arrayed Imaging Reflectometry, or “AIR”, is a label-free biosensor technique able to provide quantitative information on 10’s to 100’s of analytes simultaneously, while requiring low sample volumes (< 25 microliters) and simple instrumentation. In brief, AIR relies on the creation of a near-perfect antireflective condition on the surface of a silicon chip [3]. When target molecules bind to immobilized probes (antibodies or antigens) on the surface of the chip, this causes a disturbance in the antireflective condition, producing a change in the reflected light that quantitatively and sensitively reports the amount of the target analyte present in a sample. As a label-free technique, AIR utilizes a simple work flow involving only application of the diluted sample to the chip, incubation, and a final rinse and dry step prior to imaging. This system can be implemented using an imaging system that has no moving parts, no need for temperature control, and an estimated component cost of under $5000. Further details of the method, and its application to a broad range of targets, have been reported elsewhere [4]. In preliminary studies focused on influenza antibody detection, we examined the performance of an AIR array consisting of 5 hemagglutinins, with human samples derived from a trial of a candidate H5N1 flu vaccine [5]. We found that this array readily enabled us to profile relative antibody responses (many of which were cross-reactive) in human serum, and differentiate subjects receiving placebo from those to whom the candidate vaccine had been administered. Other groups, using labeled approaches (in which a fluorophore-tagged secondary antibody is incubated with the array post sample incubation, allowing readout via a fluorescence microarray scanner), have similarly examined the utility of influenza antigen microarrays for assessing responses to infection or vaccination [6–10]. A random peptide library has also been employed in this context [11].

Following these initial validation experiments, we sought to determine if the AIR hemagglutinin microarray could be used in the context of influenza surveillance in avian species. In particular, the label-free aspect of the AIR hemagglutinin array is attractive in this application, since the simplified workflow of such a device potentially allows for the production of field-deployable, self-contained instruments. Such devices would allow for the immediate analysis of samples in the field, rather than requiring their transport back to a centralized facility. To that end, we report here the extension of the AIR HA array to a larger number of HA isoforms, and its use in the context of analyzing avian serum samples.

## Materials and Methods

### Ethics Statement

All animal work was reviewed and approved by the University of Georgia Institution Animal Care and Use Committee (AUP#: A2013 05–021)

### Label-free substrates

Amine-reactive AIR chips were purchased from Adarza BioSystems, Inc.

### Recombinant hemagglutinins

The following recombinant hemagglutinins (Table 1) were purchased from Sino Biological, Inc., and used as supplied.

**Table 1:** Recombinant hemagglutinins used in microarray fabrication.

Hemagglutinin	Strain
H1	A/Brisbane/59/2007
H1	A/mallard/Ohio/265/1987
H2	A/Canada/720/2005
H3	A/Wisconsin/67/X-161/2005
H4	A/mallard/Ohio/657/2002
H5	A/turkey/Turkey/1/2005
H5	A/duck/NY/191255-59/2002
H6	A/northern shoveler/California/HKWF115/2007
H6	A/mallard/Ohio/217/1998
H7	A/Shanghai/2/2013
H8	A/pintail duck/Alberta/114/1979
H9	A/Hong Kong/1073/99
H10	A/mallard/Minnesota/Sg-00194/2007
H11	A/mallard/Alberta/294/1977
H12	A/green-winged teal/ALB/199/1991
B	B/Florida/4/2006

### Commercial polyclonal antisera

Hemagglutinin-specific polyclonal antisera were obtained from BEI resources. Bovine serum albumin (Fraction V, IgG and protease-free), goat anti-human IgG, goat anti-fluorescein, and mouse IgG F(c) fragment were obtained from Rockland Immunochemicals.

### HA microarray experiments

Assays were performed using modified versions of previously described AIR protocols. In brief, amine-reactive substrates as received from the manufacturer were stored in a vacuum-seal dome with Drierite prior to use. HA spotting solutions were prepared in PBS at 150 μg/ml from stock solutions, while antibody spotting solutions used as controls were prepared in PBS at 200 μg/ml from stocks. Individual antigens were arrayed on substrates by means of a piezo-electric, noncontact arrayer (Scienion S3) at a controlled humidity (~65%) using a capillary nozzle capable of producing 350 pL spots. Typically 10 spots were printed for each antigen; these were interleaved with positive (IgG) and negative (mouse IgG F(c) fragment) control spots. After printing, substrates were placed into 50 mM sodium acetate buffer (pH 5.0), followed by a dropwise addition of 100 mg/ml of BSA in PBS that resulted in a final blocking concentration of 10 mg/ml BSA solution (1%) in sodium acetate buffer. After 30 minutes in this solution, substrates received three washes with modified PBS-EDTA-Tween20 (assay wash buffer) and then were added to pre-blocked wells of a 96-well plate containing target solutions. Substrates were incubated in target solution for approximately 1 hour followed by washing with assay buffer, then phosphate buffered saline (PBS) at pH 7.4. After a brief rinse in deionized, glass-distilled water (ddH_2_O), substrates were placed on a vacuum chuck and dried with a stream of nitrogen. Substrates were either imaged immediately afterwards or stored in a vacuum-seal dome for later imaging.

### Chip imaging

Dried experimental and control arrays were imaged on a prototype AIR reflectometer (Adarza BioSystems, Inc.) with exposure times ranging from 250 ms to 1 second.

### Data analysis

Data were extracted from 16-bit images using NIH-ImageJ [12]. For individual microarray spots, median pixel intensities were reported as reflectance values (arbitrary units) and then converted to thickness (Ångstroms). This is necessary since thickness, a direct measure of the amount of material deposited on the microarray spot, and reflectance, the measured quantity, are not linearly related in the AIR method [13]. Conversion was made possible through the use of an empirical, parabolic model that characterizes the reflectance behavior of chip silicon oxide thickness at a particular integration time. The processing of data was performed in a simplified and sequential manner: intra-chip thicknesses for HA isoform spots were first normalized by adjacent negative control spots (mouse IgG F(c) fragment). This step was needed to correct for regional variations in chip topography. After normalizing all HA spots to a “baseline” negative control spot thickness, intra-chip HA isoform thicknesses were averaged (n = 5 to n = 10) and the standard deviations were obtained. This process was executed for each experimental condition (N = 1) and each HA isoform. Next, the control-exposed HA isoform means were subtracted from each of the analyte-exposed HA isoform means to produce Δ thickness values. The final errors are reported as the square root of the sum of squares between the analyte and control group standard deviations for the HA isoforms. In order to report quantitative information, we plot Δ thickness as a function of concentration.

### Field-collected serum samples

Serum samples from mallards (*Anas plathyrynchos*) were obtained from field-collected birds from northwestern Minnesota and from experimentally infected birds (*Anas plathyrynchos*, Murray McMurray Hatchery, Webster City, IA, USA) [14]. The experimental infections were done as described and serum samples were collected on day 14 post challenge [15]. Challenge viruses included A/mallard/MN/AI11-4724/2011 (H3N8), A/mallard/MN/AI11-4979/2011 (H4N6), A/mallard/MN/AI11-4982/2011 (H6N2), and A/mallard/MN/AI11-3866/2011 (H12N8); samples from these challenge experiments were tested by MN with homologous antigen. Serum samples from wild mallards were initially tested by MN against HA 1–12 as described for HA 14 in Ramey et al [16]. These samples were tested using a commercial blocking ELISA (bELISA; FlockChek AI MultiS-Screen antibody test kit; IDEXX Laboratories, Westbrook, Maine, USA) to detect antibodies against the nucleoprotein (NP). Antigens used in testing included A/mallard/MN/AI12-4823/2012 (H1N1), A/mallard/MN/AI08-2755/2008 (H2N3), A/mallard/MN/AI10-2593/2010 (H3N8), A/mallard/MN/AI10-3208/2010 (H4N6), A/mallard/MN/AI11-3933/2011 (H5N1), A/mallard/MN/AI08-2721/2008 (H6N1), A/mallard/MN/AI08-3770/2009 (H7N9), A/mallard/MN/SG-01048/2008 (H8N4), A/RUTU/DE/AI11-809/2011 (H9N2), A/mallard/MN/SG-00999/2008 (H10N7), A/mallard/MN/SG-00930/2008 (H11N9), and A/mallard/MN/SG-3285/2007 (H12N5).

## Results

### Selection of negative control conditions

In our preliminary influenza work, antibody responses were normalized relative to a negative control consisting of an identical array treated with buffer only. While effective in that study, higher sensitivity can be obtained by first correcting based on an internal negative control (here, anti-fluorescein), and then relative to a control array treated with an unreactive serum. The main issue with normalizing detection data using (m)PBS-ET buffer is that it does not allow one to distinguish between nonspecific binding from serum treatments and actual antibody-antigen interaction. Essentially, this may cause false-positive detection or a global signal offset. Substrates treated with unreactive serum epitomize ideal negative control conditions since all sera are composed of the same basic constituents [17]. Of course, such a negative-control serum must be devoid of anti-HA antibodies, suggesting a fetal source as the ideal serum matrix. While fetal bovine serum (FBS) is known to interfere with influenza infectivity, this is believed to be due to the presence of FBS constituents able to inhibit viral proteases necessary for membrane fusion [18]. Dot blots were used to confirm the absence of anti-hemagglutinin antibodies in commercial FBS.

### Validation with polyclonal antisera

**Fig 1:**
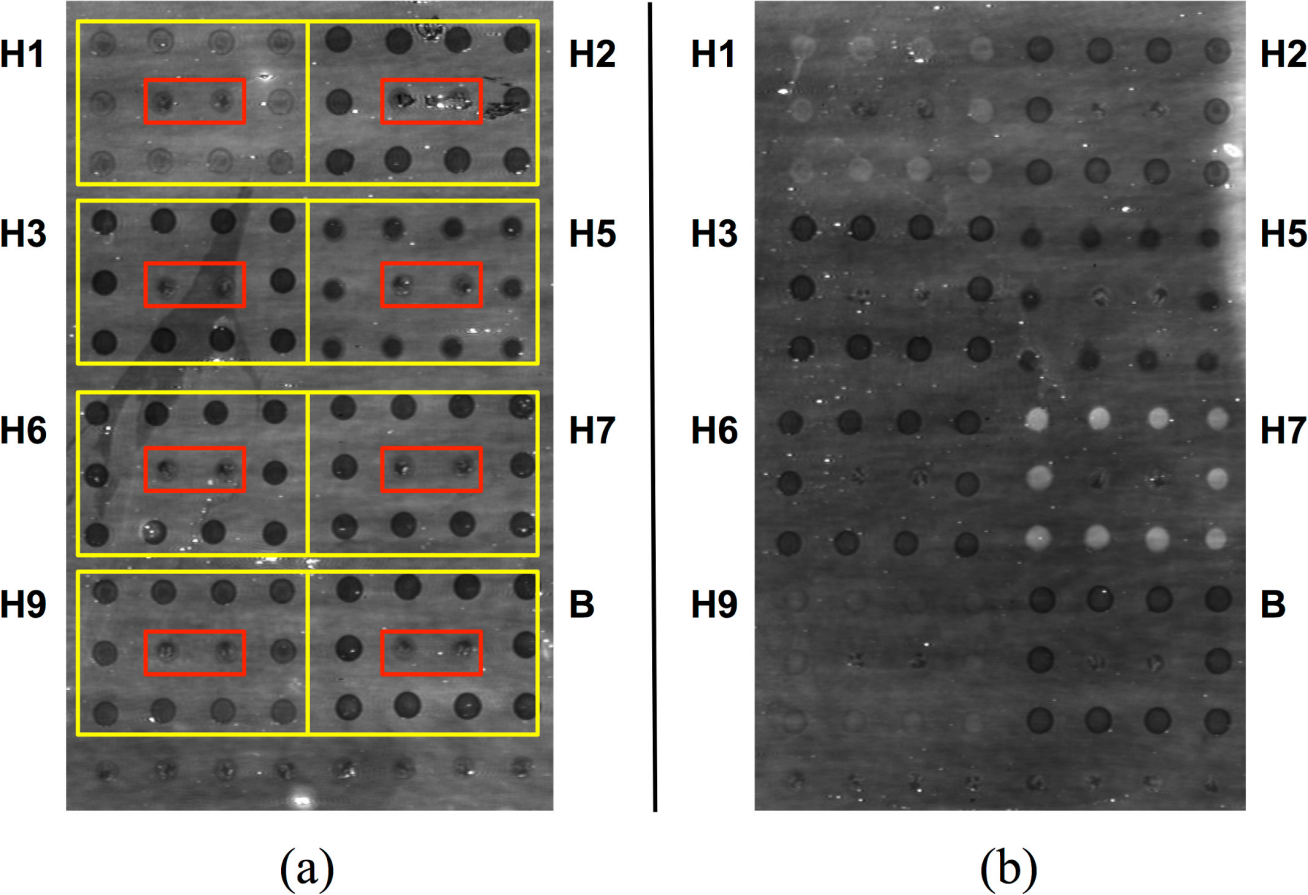
Strong responses to polyclonal anti-HA antiserum are readily observable on an AIR hemagglutinin microarray. (a) 1% BSA control. (b) Anti-H7 polyclonal antiserum (A/Netherlands/219/2003, H7N7), 1:80 dilution (1.3%) in 1% BSA. Spots showing substantially increased brightness indicate binding to immobilized H7. In both cases, antigens were arrayed in square patterns as indicated by the yellow boxes in (a); a mouse IgG Fc domain was included as negative control (red boxes). Slight differences in spot intensity in the control (a) are due to differences in deposition efficiency for different antigens or controls. Specific antigens used in these experiments are indicated in Table 2.

**Fig 2:**
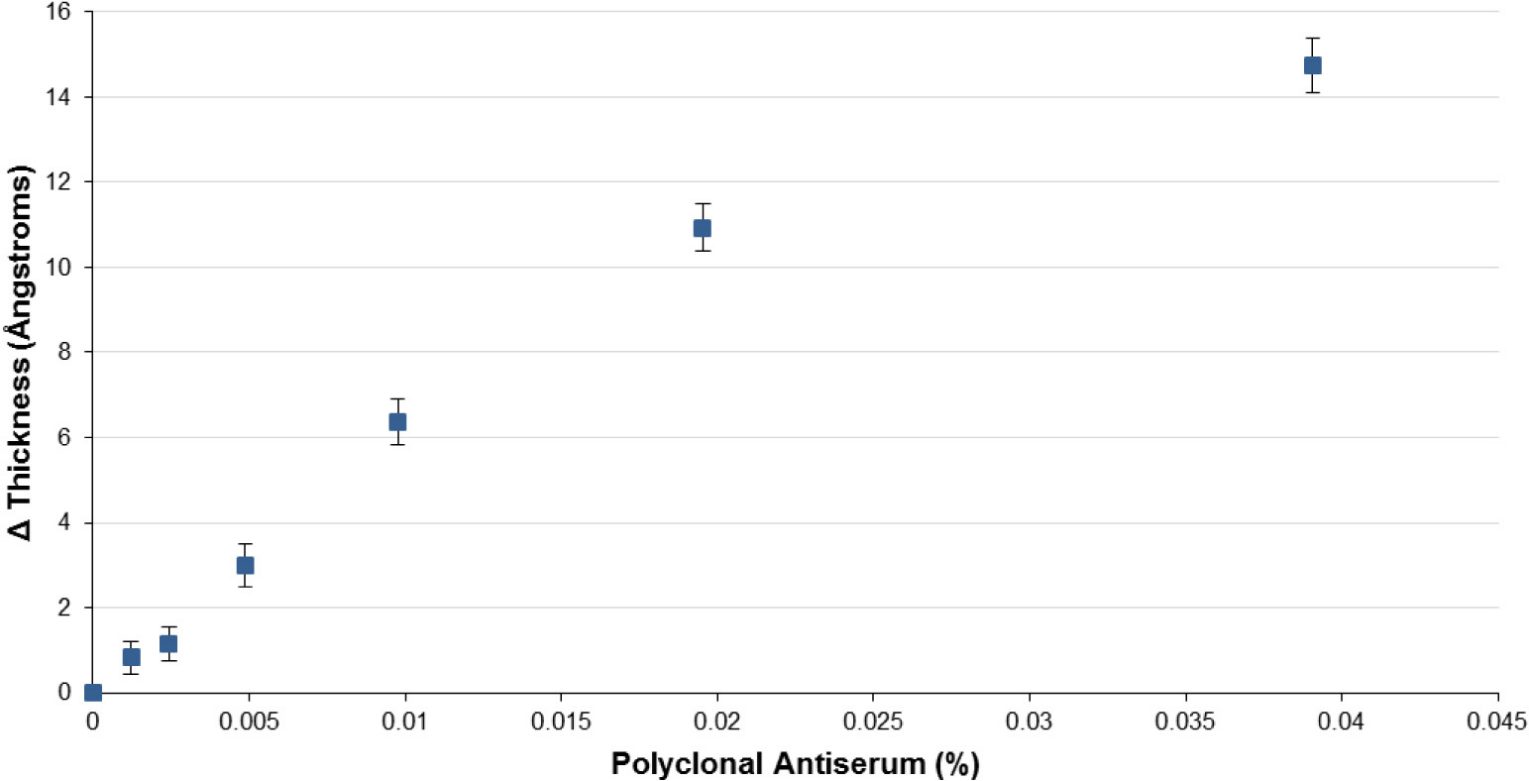
Representative array response to a dilution series of H7 polyclonal antiserum. Cross-reactivity (non-H3 response) is shown only at the highest concentration tested, but was negligible throughout. Error on each point represents the square root of the sum of squares between the analyte and control group standard deviations for the HA isoforms; N = 1, n = 10.

Following printing, the activity of selected immobilized antigens was confirmed by exposure to commercially sourced polyclonal goat antisera. This was possible only for a subset of antigens on the array, constrained by commercial availability. Dilution series for each antiserum were used to verify specificity and to assess lower limits of detection (LLOD, defined as the lowest antiserum dilution tested for which the detection signal was twice the standard deviation). In each case, the highest concentration used in the titration was that at which the relevant antigen response saturated. As one would expect, these were very different for different antigens. Fig 1 shows a representative set of array images exposed to a 1% BSA and anti-H1 antiserum. Fig 2 shows a representative dilution series for anti-H3 antiserum; dilution series for other antisera are provided in **S1 File**. Values for LLOD are provided in Table 2. Several conclusions may be drawn from these data. First, “matched” or homologous antigens responded as expected on the arrays, even though in some instances the HA used to raise the antiserum was not identical to that immobilized on the chip. Cross-reactivity in all cases was minimal, as may be readily observed from the titration shown in Fig 2. With regard to LLOD, these rely on the interplay of several factors. First, most obviously, the LLOD depends on the effective concentration of polyclonal antibody in serum. Each serum was collected following goat immunization with HA antigen and will have varying immunogenic response and, thus, IgG concentration (as well as variation in neutralizing antibody concentration, as reflected in the reported HI titer; see Table 2). Second, the LLOD depends on the analytical performance of the array, which in turn is a function of several factors, including the ability to immobilize probe molecules at a thickness near the antireflective condition, the activity of the probe molecule following immobilization, and the ability to reject nonspecific binding by non-target serum components. From Table 1, it is clear that the observed LLODs are comparable to (if not better than) measured HI titers provided by the suppliers, and within the range typically observed for an ELISA HA assay. We would not expect these LLODs to be identical, given the differences in the assay system.

**Table 2:** LLODs for polyclonal antisera as measured by AIR hemagglutinin microarray. HI titer values are as reported by the manufacturer.

Hemagglutinin	LLOD (AIR)	Antiserum antigen	Manufacturer-reported HI Titer
H1 (A/Brisbane/59/2007)	1:160	H1 (A/Brisbane/59/2007, H1N1)	1:320
H2 (A/Canada/720/2005)	1:10,240	A/Singapore/1/1957 (H2N2)	1:2,560
H3 (A/Wisconsin/67/X-161/2005)	1:8,000	H3 (A/Hong Kong/1/68, H3N2)	1:8,000
H5 (A/turkey/Turkey/1/2005)	1:2,560	H5 (A/Hong Kong/213/03, H5N1)	1:640
H6 (A/northern shoveler/California/HKWF115/2007)	1:4,800	H6 (A/teal/Hong Kong/W312/97, H6N1)	1:1,280
H7 (A/Shanghai/2/2013)	1:8,900	H7 (A/Netherlands/219/2003, H7N7)	1:1,280
H9 (A/Hong Kong/1073/99)	1:1,280	H9 (A/chicken/Hong Kong/G9/97, H9N2)	1:320
B (B/Florida/4/2006)	1:1,280	B (B/Florida/4/2006)	1:80

Detection limits are comparable to reported HI titer values derived from manufacturers’ product sheets.

### Analysis of experimental infections (mallards)

**Fig 3:**
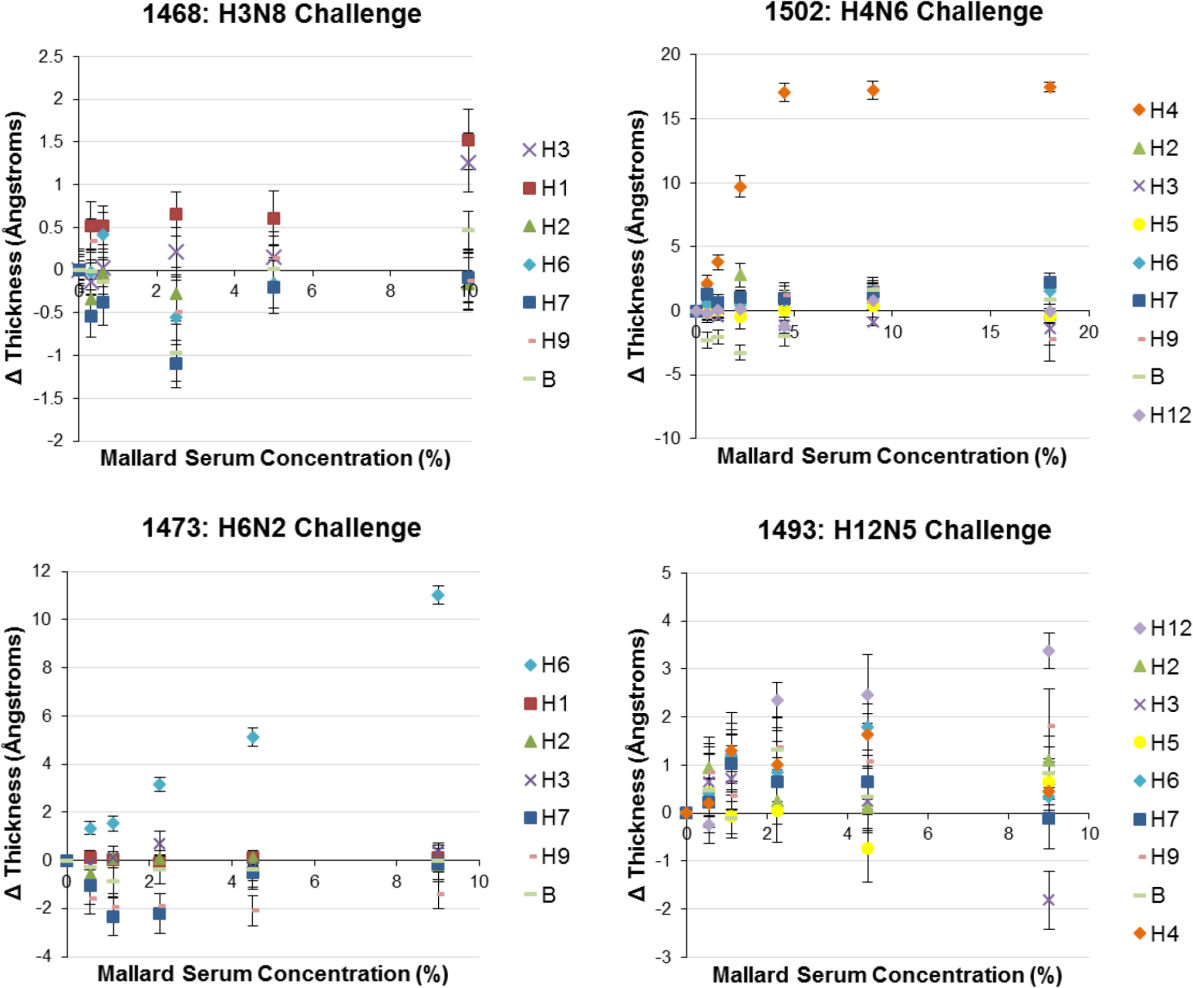
Titration plots for selected challenge samples. Samples 1502 and 1473 demonstrated highly specific and robust responses to the challenge antigen on the microarray, while samples 1468 and 1493 produced weaker and less specific responses. Error on each point represents the square root of the sum of squares between the analyte and control group standard deviations for the HA isoforms; N = 1, n = 10.

With initial testing of the array complete, we next examined serum samples from an experimental infection (challenge) of mallards. All data normalization was performed using ELISA-negative samples as a baseline. A pilot AIR study confirmed that ELISA-tested negative samples did not produce any response to HAs when normalized against an FBS control. Next, infection challenge samples (H3, H4, H6, and H12) were assayed. The dose-response relationships for all representative HAs were measured; the highest concentration reported was determined based on saturation of at least one antigen, or an 18% solution (1.8:10) if none saturated. Selected results for these experiments are shown in Fig 3; additional examples are provided in **S2 File**.

The dose-response curves for mallard challenge samples demonstrated similar behavior to the polyclonal goat antisera. Samples 1517, 1516, 1503, 1502, 1474, and 1473 showed the most robust detection responses for the particular challenge antigen of interest (Fig 3 and **S2 File**). For the majority of these samples, a specific response was detectable to a titer of at least 1:100 (1% solution). In some cases, modest thickness builds were observed for the non-challenge HAs as well, potentially reflecting cross-reactive antibody responses, or the presence of sialylated serum products able bind to the HA probes [19].

Three samples (1468, 1493, and 1492) did not show analogous subtype-specific and robust responses. Independent ELISA data indicated that samples 1493 and 1492 contained antibodies to NP protein, although subtype-specific antibodies were not detectable by MN for sample 1492. Sample 1493 had low titers of antibody (20), as measured by MN. This is consistent with data indicating that birds inoculated with H12 (1492 and 1493) did not shed virus, indicating limited viral replication. These generally consistent results suggest that this particular virus did not infect the birds and therefore did not produce a potent immunogenic response following inoculation, rather than indicating any issue with the AIR hemagglutinin microarray. MN analysis of 1468 indicated a titer of 40, and the bird was found to be shedding virus. Therefore, with respect to this sample, it is possible that the H3 in our array is antigenically distinct from the H3 virus that was used to inoculate the bird. Some of the HA responses displayed negative changes in spot thickness relative to controls. These are likely due to subtle differences in nonspecific binding for individual experimental samples relative to controls, and although they constitute a source of noise in the assay do not hinder observation of the strong positive signals. Overall, these experiments demonstrated the utility of the AIR HA microarray to effectively report on antibody titer raised in response to a viral challenge.

### Experiments with field samples (mallards)

**Fig 4:**
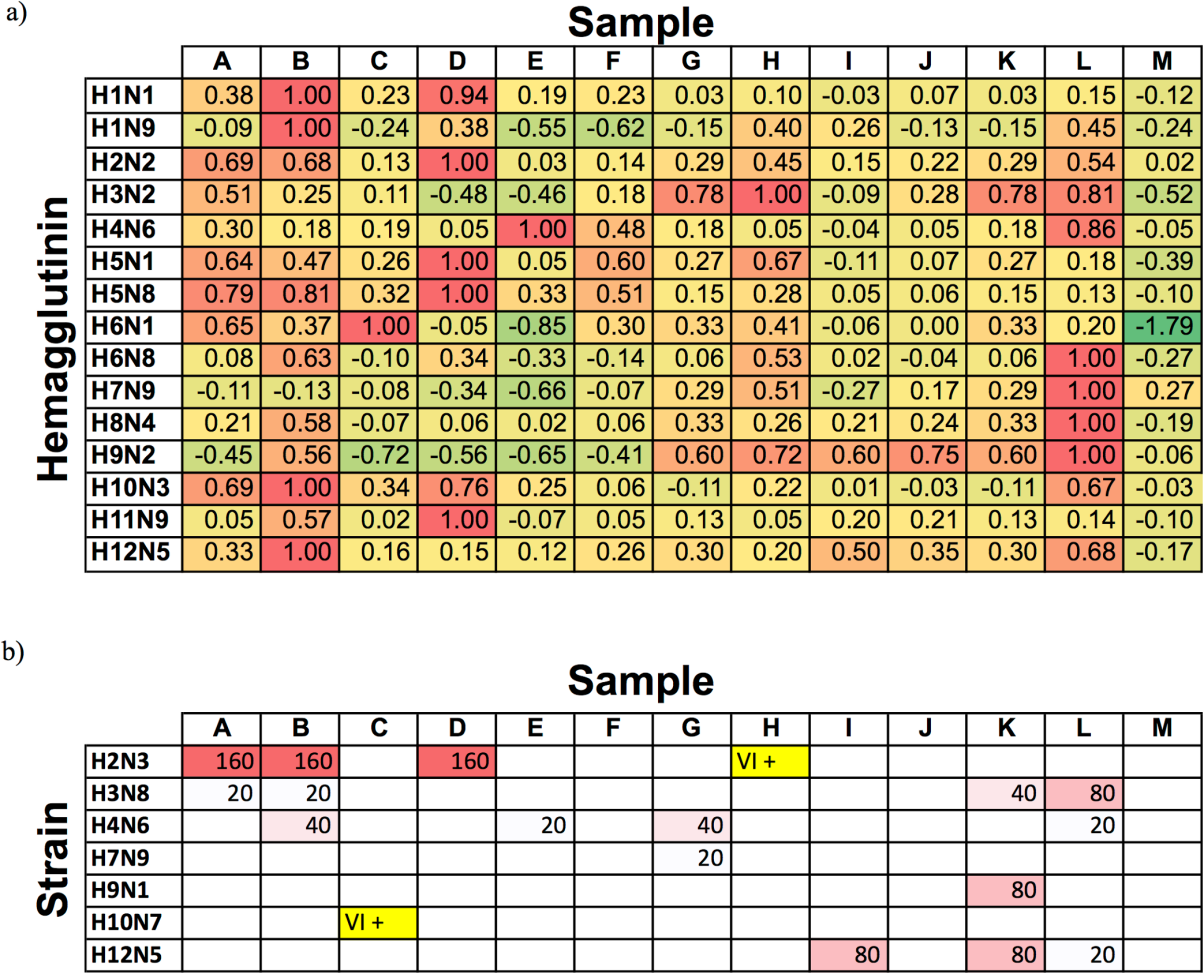
Comparison of HA microarray and selected microneutralization (MN) and virus isolation (VI) results for field samples (mallard). a) AIR microarray data. Average differences in spot thickness (normalized relative to the strongest response for each antigen) for chips treated with 18% field serum are reported relative to control. Color coding (green = low to red = high) is scaled relative to this maximum response. b) MN results (numbers indicate antibody titer); two samples highlighted in yellow tested positive by VI. Empty cells indicate an antibody titer of < 20.

To further assess the utility of the HA array for analyzing influenza responses of natural infections in avian serum, we tested 13 serum samples taken from mallards in the field. These samples were prescreened for antibodies to influenza nucleoprotein using a commercial blocking ELISA (bELISA) assay, with 10 of the 13 yielding a positive signal. Responses for these samples from the AIR hemagglutinin array are shown in Fig 4 and **S1 Fig**. As is evident from the figure, the AIR array provides a rich set of information that is complementary to (but not identical with) serological data obtained using conventional methods (Fig 4B; as validated subtype-specific avian serum ELISA assays are only available for H5 and H7, we instead compared to MN and virus isolation assays). Responses for H2 virus correlated particularly well, with samples A, B, and D showing a dramatic thickness build on the AIR array as well as strong MN titers, despite the use of an H2N2 antigen on the AIR array and an H2N3 virus in the MN assay. All-negative samples (F, J, M) are also in good agreement. As an example of differences between the assay formats, sample L produced a response consistent with significant concentrations of antibodies to H2, H3, H4, H6, H7, H8, H9, and H12, while MN assays were positive for only H3, H4, and H12. As in our previous work with human samples, it is possible that differences in response are indicative of the presence of non-neutralizing antibodies, or of cross-reactivity with immobilized hemagglutinins.

## Discussion and Conclusions

As a primary reservoir of type A influenza viruses, understanding the prevalence of various subtypes as well as exposure history in avian species is of considerable importance [20]. Ideally, efforts along these lines would involve serological assessment in the field, as rapidly available results could be used to guide further collections during a study. Currently, however, the complexity of current “gold-standard” serological methods makes this impossible. In this report, we have shown that a hemagglutinin microarray constructed on the Arrayed Imaging Reflectometry (AIR) platform can provide multiplex information about anti-HA antibody titers in avian serum. While not identical to results obtained via MN, these data are complementary to and extend results obtained via standard techniques. Results obtained from the AIR platform correctly identified subtypes used to experimentally infect mallards. For field samples, array data also provided a serologic profile that in most cases mirrored MN and virus isolation results from the test samples and reflected the extensive influenza subtype diversity that is annually present at this field site [14]. Importantly, as a label-free methodology with a simple workflow and low sample volume requirement (≤ 20 microliters), we anticipate that the AIR HA microarray is ideally suited to field applications. The fact that the AIR microarray is not species-specific is also likely to be useful, as obtaining secondary antibodies to specific wild species can be challenging. Thus, samples from many potential avian and mammalian hosts may be evaluated without modification of the assay system. Since the current array uses only a small fraction of the total AIR chip surface area, expansion to additional HA variants (or fragments of HA) is trivial. Efforts along those lines are currently in progress.

## Supporting Information

S1 Fig Alternative format of Fig 4; raw thickness changes for each antigen. (PDF)

S1 File Dilution series for H1, H2, H3, H5, H6, H7, H9, and B polyclonal antisera. H1 polyclonal antiserum titration (Figure A). H2 polyclonal antiserum titration (Figure B). H3 polyclonal antiserum titration (Figure C). H5 polyclonal antiserum titration (Figure D). H6 polyclonal antiserum titration (Figure E). H7 polyclonal antiserum titration (Figure F). H9 polyclonal antiserum titration (Figure G). B polyclonal antiserum titration (Figure H).
(PDF)

S2 File Results for five additional mallard challenge samples. (PDF)
