# Supplementary material for: A Multiplex Label-Free Approach to Avian Influenza Surveillance and Serology
Source: PLoS One. 2015 Aug 4;10(8):e0134484. doi: 10.1371/journal.pone.0134484 (PMC4524619; doi:10.1371/journal.pone.0134484)
Supplement: S1 Fig — (PDF) [file pone.0134484.s001.pdf]

Field Sample Responses

The Figure below is an alternative version of Figure 4 from the main text. Here, the actual response (thickness in Ångstroms) for each antigen is presented for chips treated with 18% field serum are reported relative to control. Color coding (blue = low to red = high) is scaled by antigen.

Sample

Hemagglutinin	A	B	C	D	E	F	G	H	I	J	K	L	M
**H1N1**	3.12	8.14	1.89	7.65	1.57	1.84	0.25	0.84	-0.27	0.58	0.25	1.21	-0.98
**H1N9**	-0.50	5.29	-1.29	1.99	-2.91	-3.25	-0.78	2.10	1.36	-0.71	-0.78	2.37	-1.28
**H2N2**	5.68	5.56	1.10	*8.24*	0.28	1.16	2.35	3.67	1.27	1.80	2.35	4.43	0.20
**H3N2**	0.70	0.34	0.15	-0.66	-0.64	0.25	1.08	1.38	-0.13	0.38	1.08	1.12	-0.72
**H4N6**	3.20	1.90	2.04	0.50	*10.64*	5.12	1.86	0.57	-0.44	0.50	1.86	*9.19*	-0.50
**H5N1**	1.86	1.36	0.76	2.89	0.15	1.72	0.79	1.93	-0.32	0.21	0.79	0.53	-1.14
**H5N8**	6.17	6.33	2.50	7.81	2.55	4.00	1.14	2.19	0.40	0.50	1.14	1.00	-0.79
**H6N1**	0.47	0.27	0.73	-0.04	-0.62	0.22	0.24	0.30	-0.04	0.00	0.24	0.15	-1.31
**H6N8**	0.33	2.76	-0.44	1.48	-1.43	-0.59	0.24	2.32	0.08	-0.19	0.24	4.35	-1.18
**H7N9**	-0.20	-0.23	-0.14	-0.62	-1.22	-0.13	0.53	0.94	-0.49	0.30	0.53	1.84	0.50
**H8N4**	1.10	3.00	-0.34	0.30	0.12	0.30	1.70	1.32	1.10	1.24	1.70	5.15	-0.96
**H9N2**	-1.44	1.80	-2.30	-1.80	-2.08	-1.33	1.92	2.32	1.92	2.39	1.92	3.20	-0.19
**H10N3**	4.74	*6.88*	2.37	*5.24*	1.74	0.39	-0.78	1.54	0.08	-0.20	-0.78	4.63	-0.20
**H11N9**	0.47	4.91	0.17	*8.67*	-0.63	0.42	1.10	0.44	1.72	1.85	1.10	1.18	-0.87
**H12N5**	2.32	6.97	1.14	1.07	0.86	1.80	2.06	1.42	3.47	2.44	2.06	4.76	-1.18

S1 Fig
