# Supplementary material for: A Multiplex Label-Free Approach to Avian Influenza Surveillance and Serology
Source: PLoS One. 2015 Aug 4;10(8):e0134484. doi: 10.1371/journal.pone.0134484 (PMC4524619; doi:10.1371/journal.pone.0134484)
Supplement: S1 File — H1 polyclonal antiserum titration (Figure A). H2 polyclonal antiserum titration (Figure B). H3 polyclonal antiserum titration (Figure C). H5 polyclonal antiserum titration (Figure D). H6 polyclonal antiserum titration (Figure E). H7 polyclonal antiserum titration (Figure F). H9 polyclonal antiserum titration (Figure G). B polyclonal antiserum titration (Figure H). (PDF) [file pone.0134484.s002.pdf]

Polyclonal antiserum titrations

Figs A-H. Representative antigen-specific array response(s) to dilution series of H1, H2, H3, H5, H6, H7, H9, and B polyclonal antisera, respectively. Cross-reactivity was negligible throughout, and therefore, not shown except on Figure C as a representative example. Error on each point represents the square root of the sum of squares between the analyte and control group standard deviations for the HA isoforms; N=1, n=10.

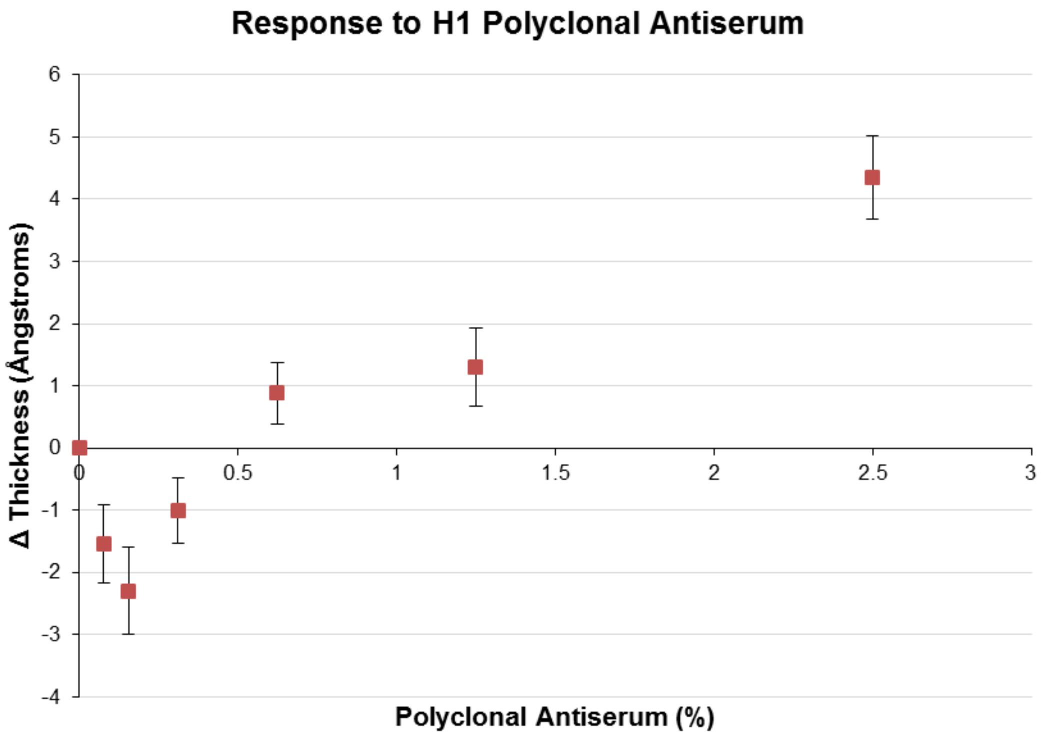

Figure A: H1 Polyclonal Antiserum Titration.

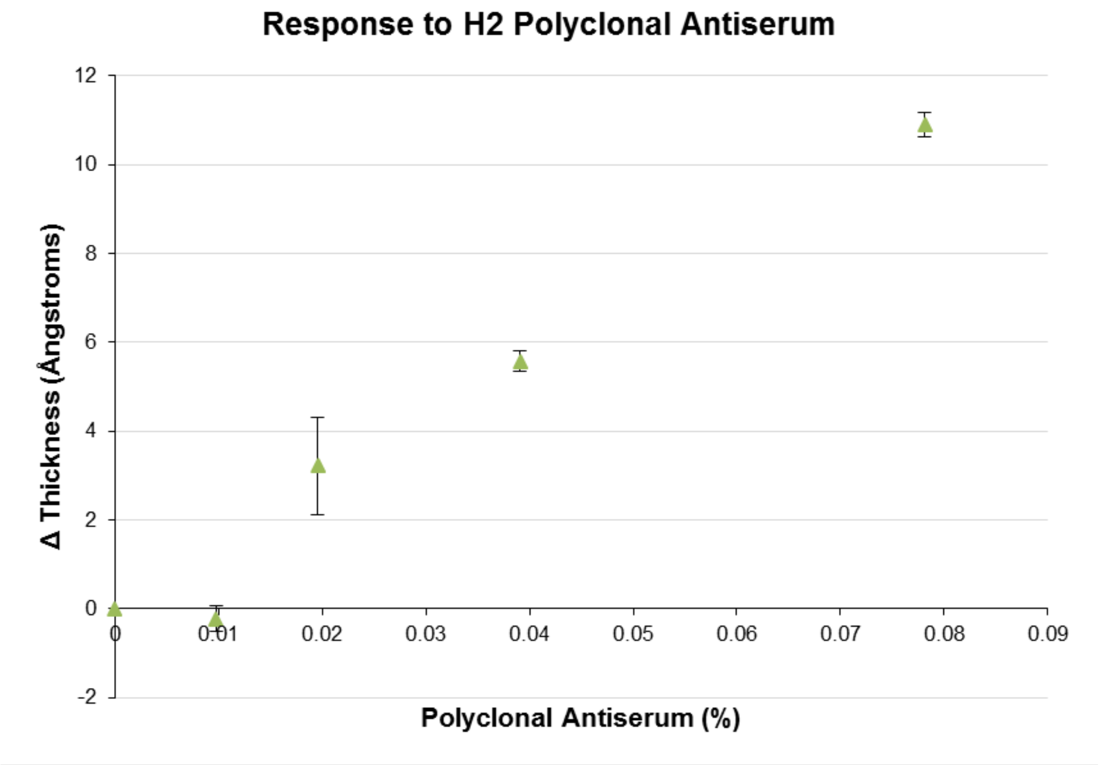

Figure B: H2 Polyclonal Antiserum Titration.

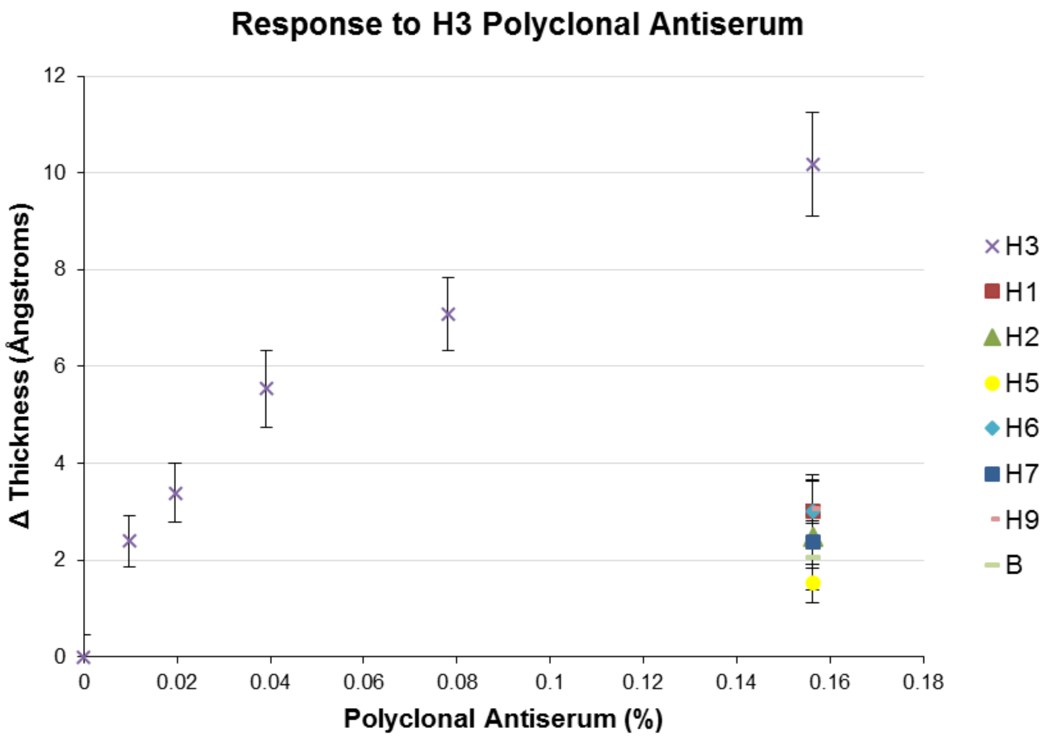

Figure C: H3 Polyclonal Antiserum Titration.

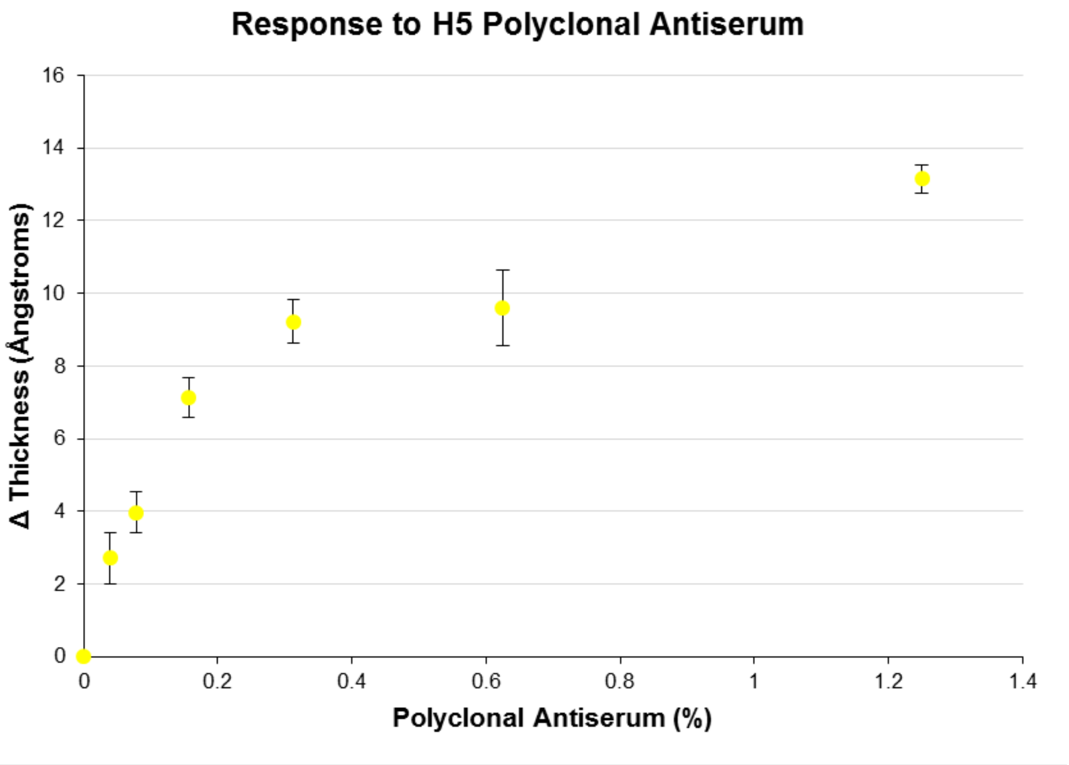

Figure D: H5 Polyclonal Antiserum Titration.

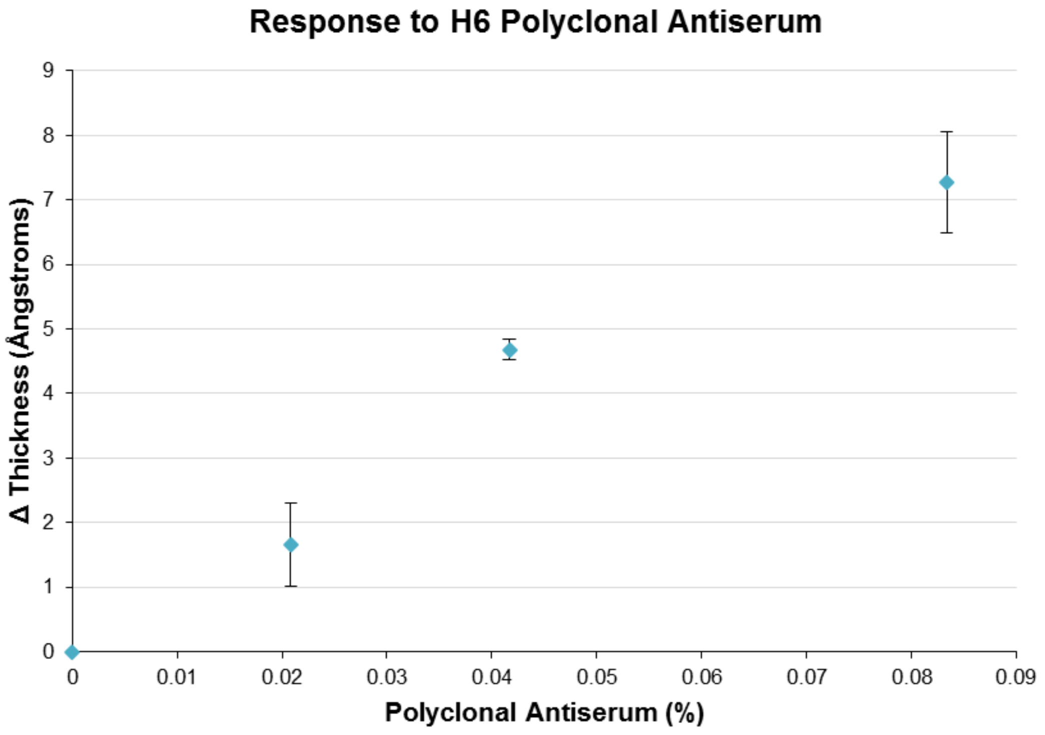

Figure E: H6 Polyclonal Antiserum Titration.

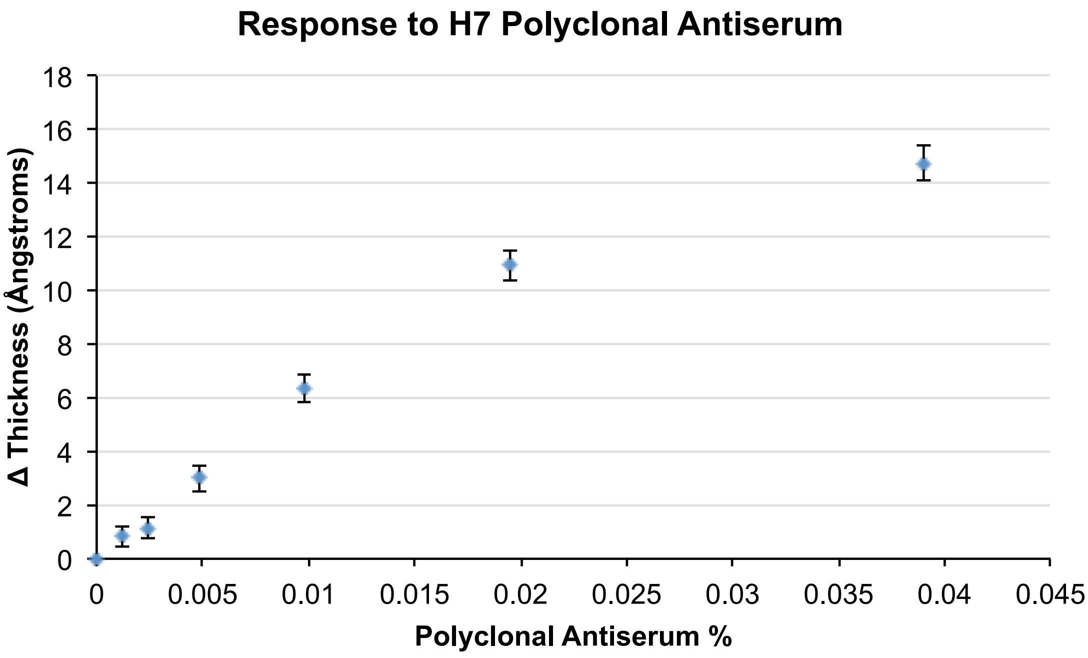

Figure F: H7 Polyclonal Antiserum Titration.

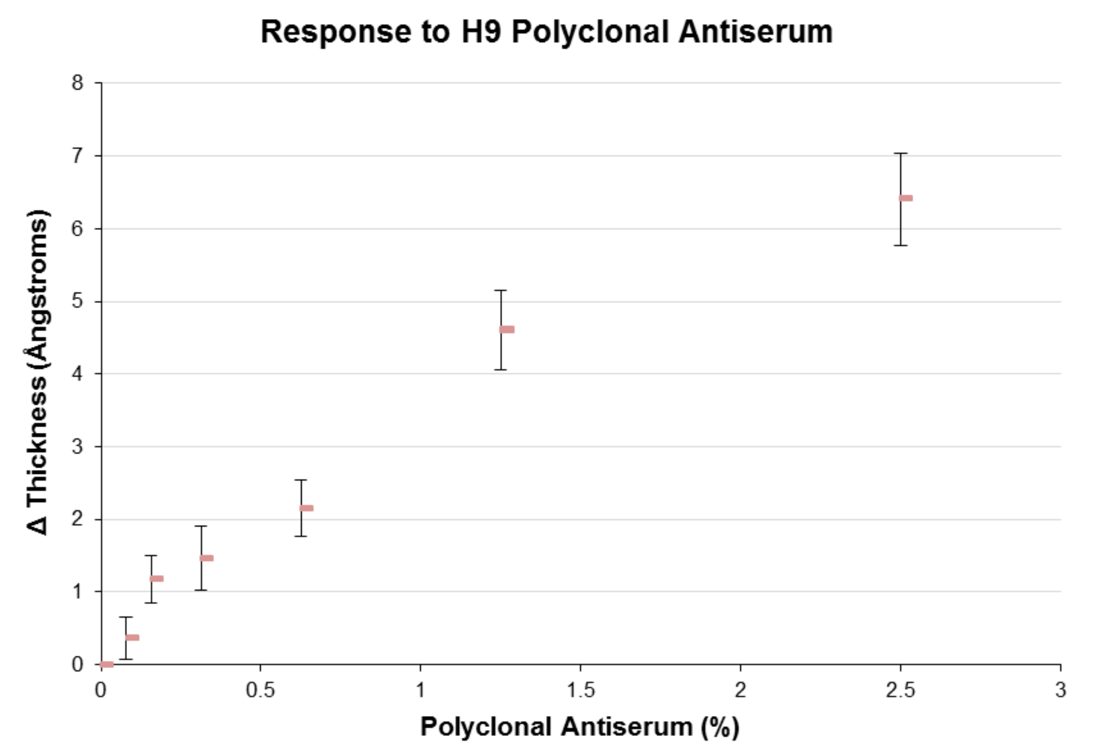

Figure G: H9 Polyclonal Antiserum Titration.

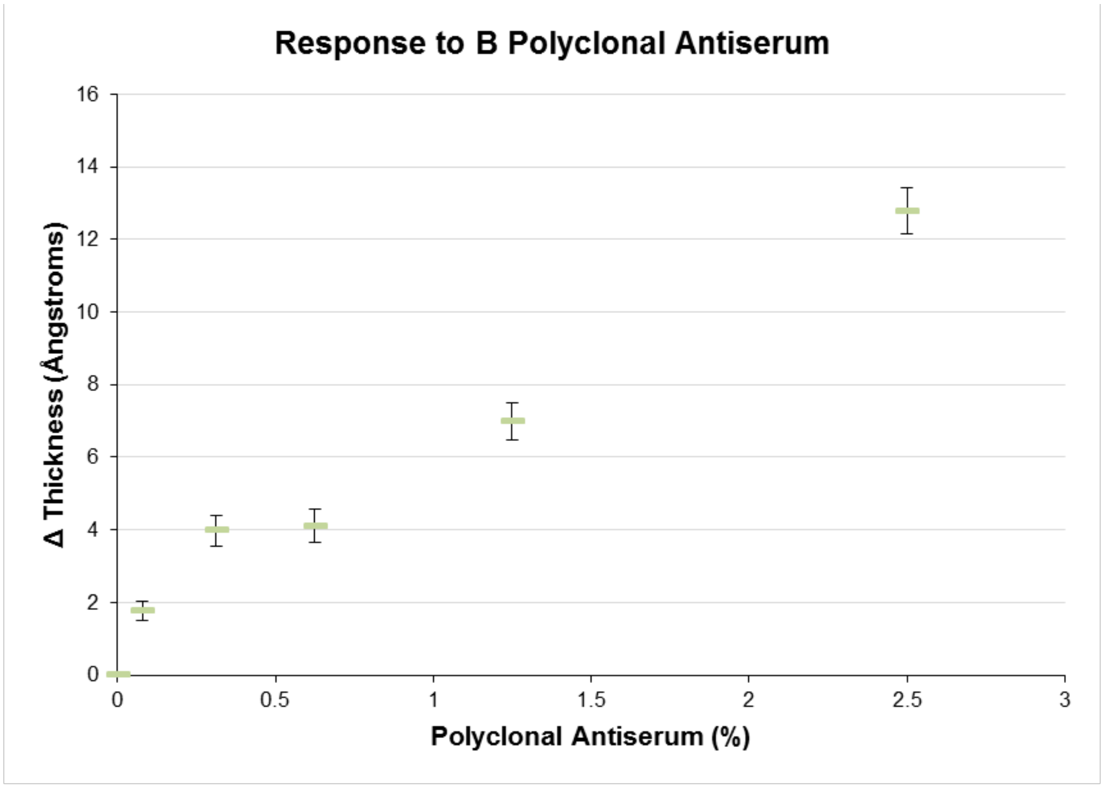

Figure H: Type B Polyclonal Antiserum Titration.
