# Supplementary material for: A Multiplex Label-Free Approach to Avian Influenza Surveillance and Serology
Source: PLoS One. 2015 Aug 4;10(8):e0134484. doi: 10.1371/journal.pone.0134484 (PMC4524619; doi:10.1371/journal.pone.0134484)
Supplement: S2 File — (PDF) [file pone.0134484.s003.pdf]

Mallard challenge plots

Figs A-E. Titration plots for remaining challenge samples. Samples 1474, 1503, 1516 and 1517 demonstrated highly specific and robust responses to the challenge antigen on the microarray, while Sample 1492 produced weaker and less specific responses. Error on each point represents the square root of the sum of squares between the analyte and control group standard deviations for the HA isoforms; N=1, n=10.

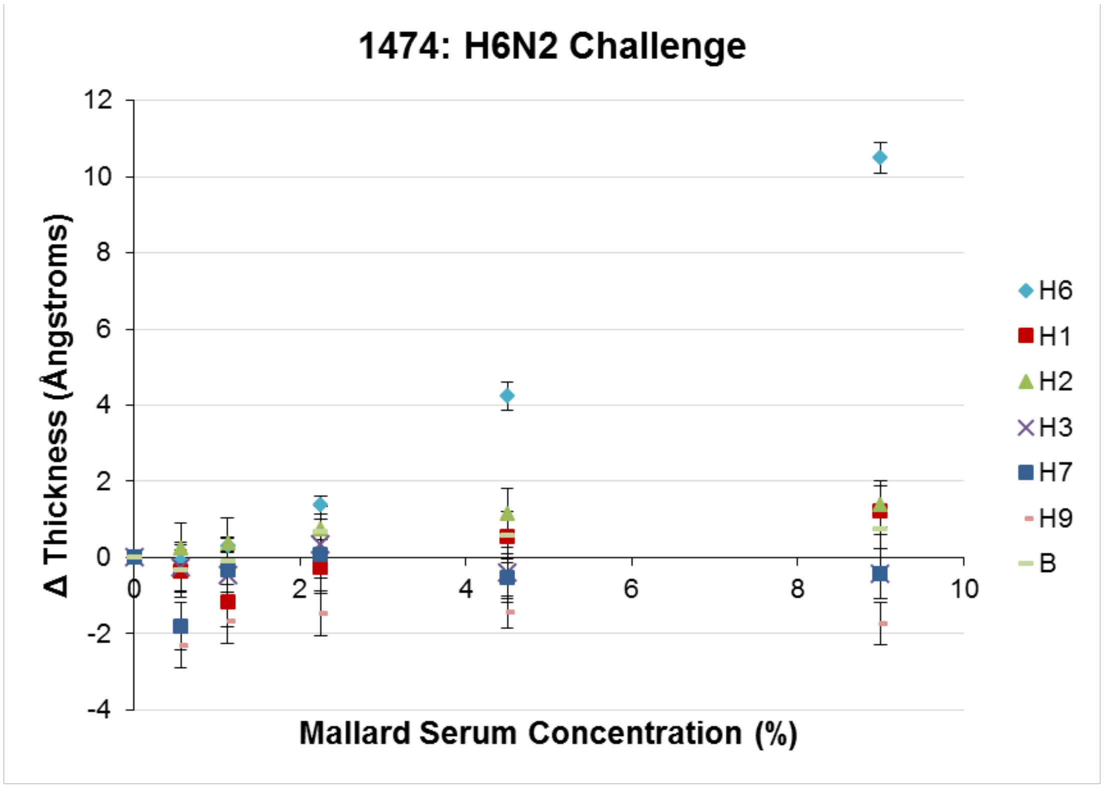

Figure A. Mallard Challenge Sample 1474.

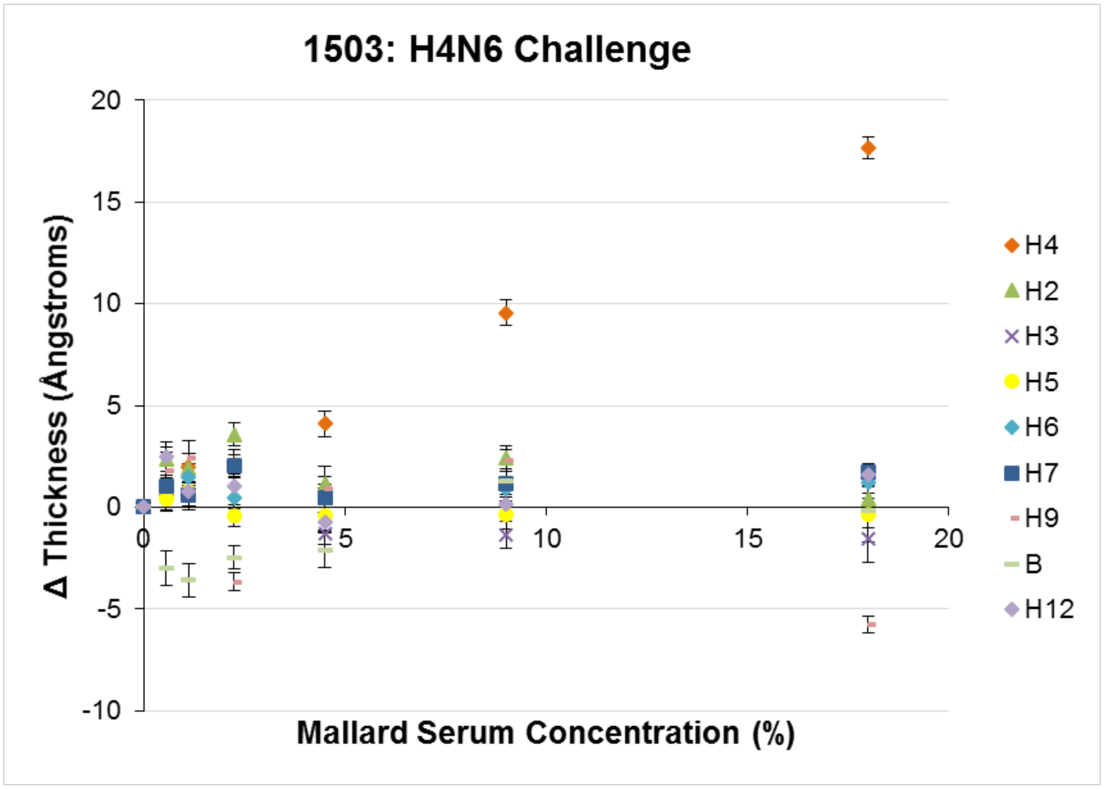

Figure B. Mallard Challenge Sample 1503.

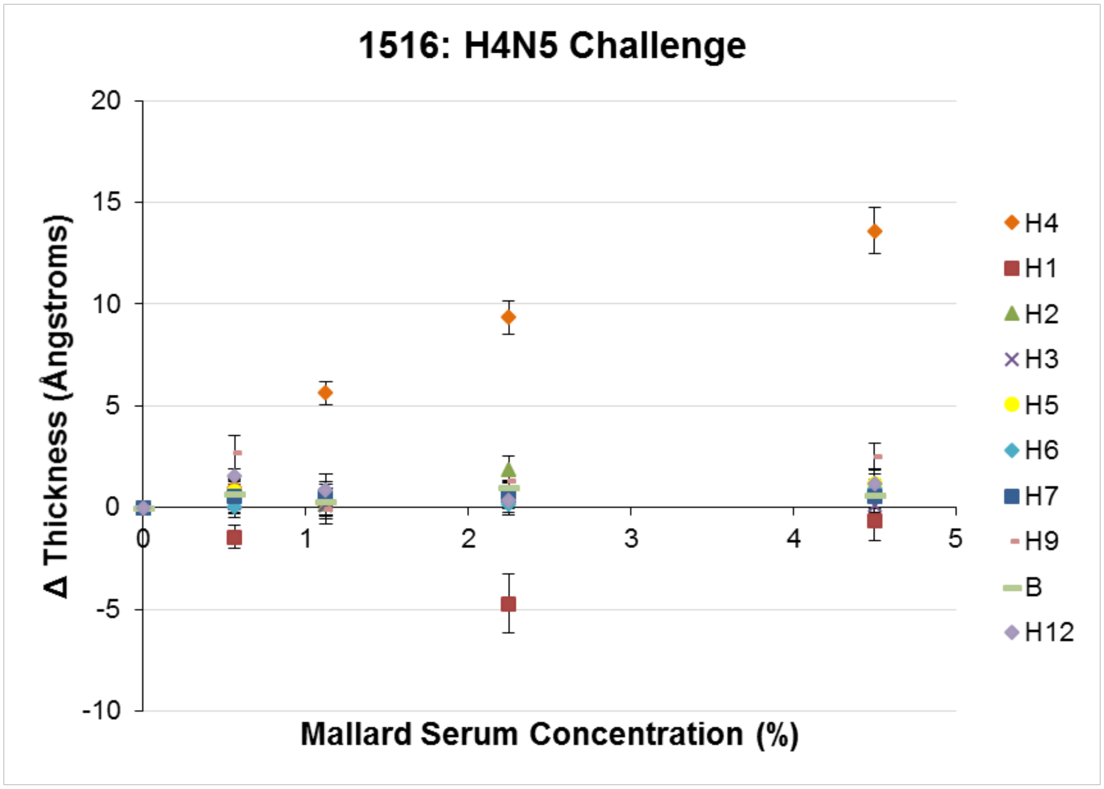

Figure C. Mallard Challenge Sample 1516.

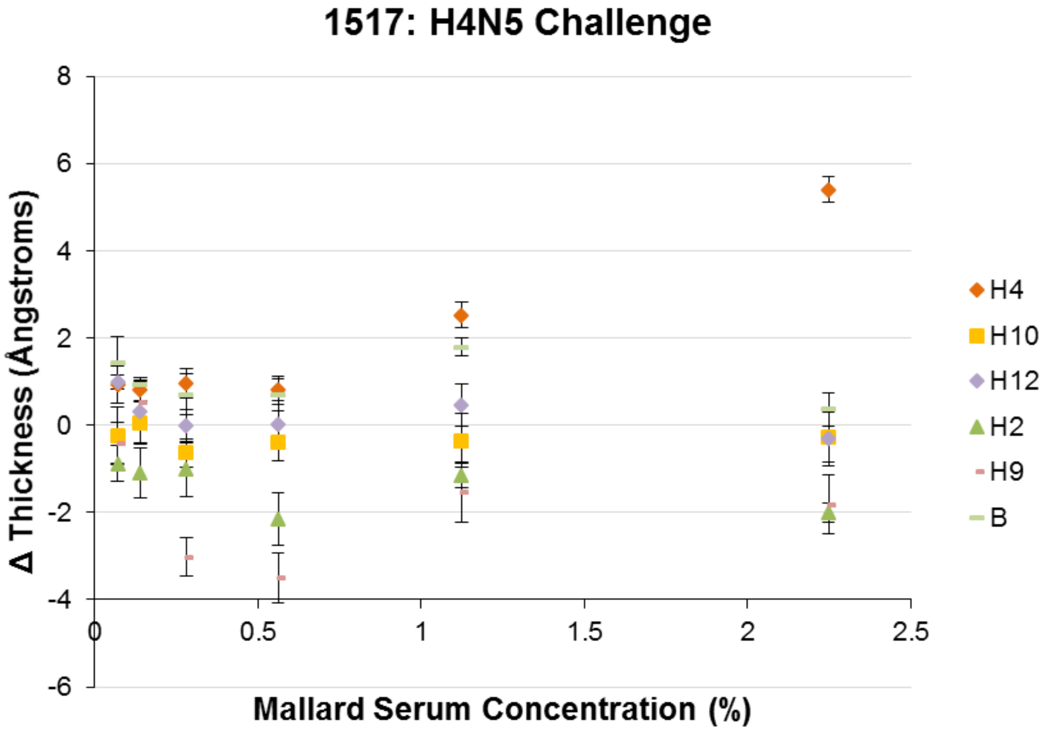

Figure D. Mallard Challenge Sample 1517.

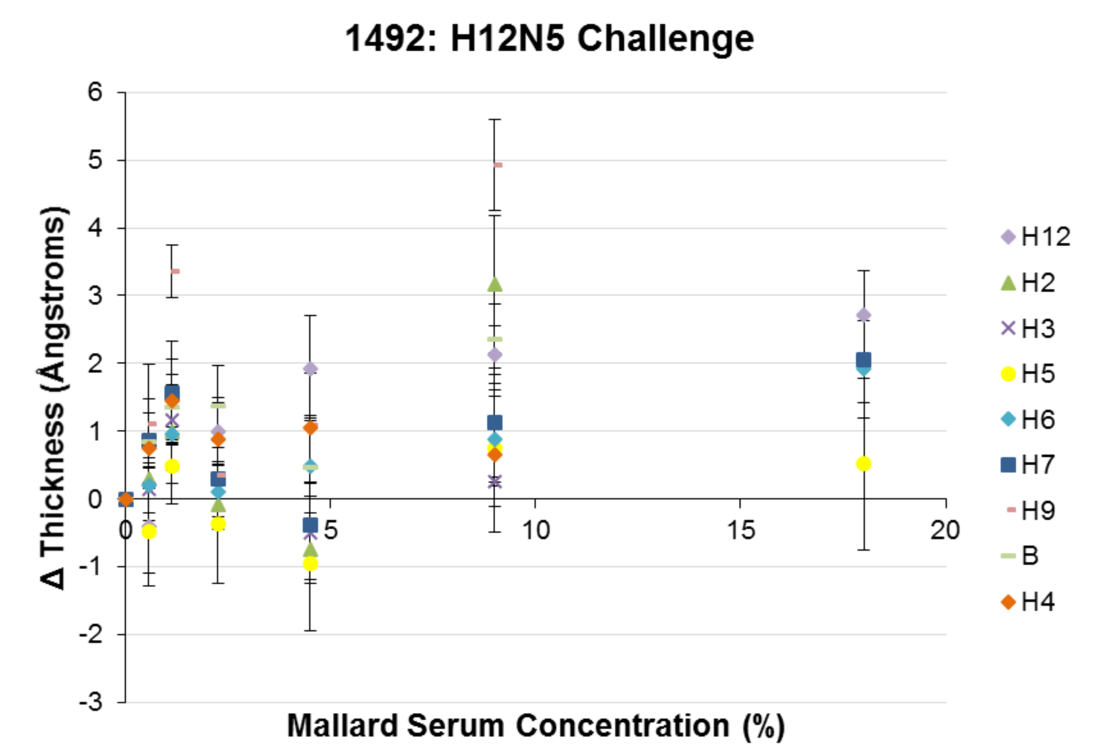

Figure E. Mallard Challenge Sample 1492.
